# Pyorrhea

**Published:** 1906-02

**Authors:** E. R. Vaughan

**Affiliations:** Denver, Colo.


### PYORRHEA.

By Dr. E. R. Vaughan, Denver, Colo.

The treatment and cure of pyorrhea, I believe, are within the reach of every progressive dentist who is willing to work. The tired operator is advised to shun the work, as it is an operation not fitted to a lazy man's temperament. The most absolute thoroughness must mark every stage.

Pyorrhea is a result, not a cause, a result of neglect, always. Some little spot in the dental arch is overlooked and a minute nodule of calculus is deposited. This causes no discomfort and so remains, becoming a source of infection, a nucleus from which disease spreads from tooth to tooth until the entire denture is involved.

When such a case presents there is only one course—thorough removal of the deposit, followed by continued and careful polishing.

As to the instruments for this work, each operator must be his own judge, but he must have them so shaped that he can reach every surface of every tooth. Personally I do more work with different forms of spoon excavators, together with some form of scaler which I may have devised from time to time as the case required, than with any of the set forms of scalers in the market.

Begin at the center of the arch and work back carefully, removing every particle of deposit from each tooth before proceeding to the next. No matter how deep the pocket or how tightly the tartar adheres, it must be removed, or failure will be the result. Educate your fingers so that when your instrument comes in contact with a foreign body, you can at once detect it. In this skill your success will lie, as you must rely wholly on the sense of touch in your treatment. Do not give long sittings. An hour is enough for any patient's endurance.

After each sitting, swab the gums treated at that time with lactic acid, full strength, taking particular care to work it down deep into the pockets. This acts as an antiseptic and as an astringent and also assists in dissolving any particles of calculus that may have been overlooked.

Then polish, polish, polish and continue to polish if you wish success. For this purpose I use to some extent the brushes and soft rubber points with the engine, but for the approximal surfaces the deep pockets and the inaccessible places, a piece of orange wood in a suitable porte-polisher and held by the hand, I find more satisfactory. Be sure that every exposed portion of each tooth is fully polished before you pass to the next.

In all cases when the teeth are loose in their sockets, absolute rigidity must be obtained by some mechanical appliance. In my hands the permanent splint which I exhibited at the meeting last fall has filled the bill precisely. I have used it in about forty cases and it has become my mainstay in this class of work.

During and after treatment I direct my patient to use some antiseptic wash.

My patients are directed to return to me every thirty days for examination, and if at that time I find any deposit I remove it, and always, whether the deposit appears or not, I give the teeth a thorough polishing.

In conclusion I wish to make the assertion that pyorrhea is not a constitutional disease, and that all the drugs in Christendom will have no more effect than so much water. Pyorrhea is only curable by local treatment, and without constant care at the outset, and occasional care later on, it is bound to recur. Any dentist of ordinary ability can treat and cure the disease, provided he is thorough and conscientious in his work, and has the co-operation of his patient. Last, but not least, his patient will pay him a larger fee, both in cash and gratitude, than for any other operation he is called on to perform.—*Items.*

